# An optimal designed experiment for the alkaline hydrolysis of feather keratin

**DOI:** 10.1007/s11356-021-17649-2

**Published:** 2021-11-25

**Authors:** Małgorzata Dąbrowska, Agata Sommer, Izabela Sinkiewicz, Antoni Taraszkiewicz, Hanna Staroszczyk

**Affiliations:** Department of Food Chemistry, Technology and Biotechnology, Gdansk University of Technology, G. Narutowicza 11/12, 80-233 Gdańsk, Poland

**Keywords:** Feather waste, Keratin, Alkaline hydrolysis, Optimization

## Abstract

Feathers, burdensome waste from the poultry industry, can be a cheap source of keratin, a protein with excellent physicochemical, biological, and mechanical properties. Acid and alkaline hydrolyses are usually adopted for isolation of keratin from its natural resources. This study aimed at assessing the statistically significant effect of input variables in the alkaline hydrolysis of keratin from chicken feathers on the process yield and on the molecular weight of peptides obtained. The effect of the volume ratio of 1M NaOH to the feathers’ mass, the hydrolysis time, and the shaking speed of the reaction mixture on the process yield were analyzed. The use of statistical analysis at the design step of experiment allowed reducing the trial number from 27 to 9. Among the input variables analyzed, only the volume ratio of 1M NaOH to the feathers’ mass had a significant effect on the process yield, while none of them significantly affected the molecular weight of the peptides obtained. All hydrolysates were dominated by two peptides’ fractions, with molecular weights of ca. 130 and 250 kDa, and mixture of many peptides of weight close to 10 kDa and smaller. Alkaline hydrolysis of feather keratin yielded protein hydrolysates soluble over a wide pH range.

## Introduction

Over the past 20 years, global plastic production has increased by around 260 million tons, ultimately reaching 359 million tons annually (PlasticsEurope 2020). Disposal of these materials is an increasing environmental challenge. During plastic waste decomposition, many toxic compounds migrate into the soil and groundwater, contaminating them and eventually killing the ecosystems that live there (Vethaak and Leslie 2016; Haider et al. 2019). When plastics are used for food packaging, the harmful compounds can be released into food, and hence, into consumer organisms, where they can bioaccumulate (Vethaak and Leslie 2016). Despite the negative aspects of using plastic, there are also many advantages of these materials, such as the resistance to mechanical damage, the low water vapor permeability, and the easy and cheap production. It is difficult to obtain a competitive biodegradable material with such good performance characteristics as synthetic materials.

The raw materials for the production of biodegradable materials can be proteins and polysaccharides, including protein- and polysaccharide-rich biomass resources (Schrooyen et al. 2000). For example, feathers from the poultry industry which account for ca. 10% of the total weight of a mature chicken are 90% keratin (Cameron et al. 2003; Singh et al. 2017; Ciechanska et al. 2014). According to the periodic report published by the US Department of Agriculture, in the April 2021, over 100 million chickens were slaughtered annually, resulting in about 100 million tons of problematic feather waste (USDA, 2021). Thus, the use of waste biomass for the production of biodegradable materials is beneficial from both an economic and an environmental point of view (Singh et al. 2017; Ciechanska et al. 2014).

Keratin is a natural polymer with a structure and properties strikingly similar to synthetic polymers. Like the latter, keratin is a cross-linked macromolecule containing plasticizers and stabilizers (Hill et al. 2010). Due to a high number of hydrophobic amino acids and a highly cross-linked structure, keratin-based materials can not only be biodegradable but can also exhibit low water vapor permeability, low water absorption, and high resistance to mechanical damage, which has been difficult to achieve so far in the production of materials from raw materials of natural origin (Schrooyen et al. 2000; Dou et al. 2016; Reddy et al. 2013). Such keratin materials can contribute to the elimination of disadvantages of currently produced biodegradable protein-based materials (Schrooyen et al. 2000; Dou et al. 2016; Reddy et al. 2013), and to lower the use of synthetic polymeric materials and, consequently, to reduce the amount of problematic non-biodegradable waste.

Keratin belongs to a heterogenous group of hydrophilic proteins insoluble in neither polar nor non-polar solvents, which are made in vertebral epithelial cells, forming animal fibers. Keratin products are durable and resistant to both physicochemical, biological, and mechanical external factors. These unique properties are related to the high content of cysteine residues, a sulfur amino acid, which allows the formation of intra- and intermolecular disulfide bridges between amino acids located in two different peptide chains, giving the keratin materials a hard and cross-linked structure (Cameron et al. 2003; Singh et al. 2017; Wang et al. 2016a). Feather keratin, with a molecular weight of ca. 10 kDa, consisting of 70–75% α-helix and 25–30% β-sheet structure, is classified as β-keratins characterized by a higher sulfur content than keratin from other sources due to a large proportion of cysteine residues in their primary sequences (Cameron et al. 2003; Singh et al. 2017; Ciechanska et al. 2014; Schrooyen et al. 2001). This increases the frequency of cross-linking keratin via disulfide linkages, thereby enhancing the hardness, toughness, and strength of the feather structure (Cameron et al. 2003; Singh et al. 2017; Wang et al. 2016b).

Due to a high content of hydrophobic amino acids, a large number of intermolecular disulfide bridges, and a densely packed structure, keratin raw materials are insoluble in traditional protein solvents. However, the key aspect of keratin isolation is to bring it into the dissolved form (Singh et al. 2017; Schrooyen et al. 2001). Redox reactions and hydrolysis with both acid and alkaline reagents belong to the main chemical methods of keratin dissolution (Chooi Wei Cheong et al. 2018; Hill et al. 2010; Kurbanoglu and Kurbanoglu 2007; Vineis et al. 2021). The reduction method with the use of 2 mercaptoethanol and urea allows for a high degree of keratin isolation from the raw materials, while limiting hydrolysis degree of peptide bonds. However, reducing reagents are highly toxic to the environment and human health, and they are too expensive to be used on a technical scale (Schrooyen et al. 2001; Nakamura et al. 2002). The properties of keratin hydrolysates depend on the pH, temperature, and reaction time (Coward-Kelly et al. 2006a, 2006b). The use of acid hydrolysis allows to a high protein extraction yield, but can result in the strong hydrolysis of peptide bond which lead to high protein fragmentation and loss of some amino acids such as tryptophan, methionine, and histidine (Liu et al. 1989). Although disulfide bridges and peptide bonds are also broken during alkaline hydrolysis, it can result in a mixture of all amino acids present in the native protein (Singh et al. 2017; Coward-Kelly et al. 2006a, 2006b; Gousterova et al. 2005; Nagai and Nishikawa 1970; Song et al. 2013; Tsuda and Nomura 2014). An increased reaction temperature and a high concentration of alkali used lead to an increased protein yield but cause its strong fragmentation, bringing about low-molecular-weight protein fractions. Unfortunately, such proteins do not show film-forming properties, unlike e.g. collagen and gelatin characterized by a high molecular weight, ca. 150–300 kDa (Abraham et al. 2008; Gómez-Guillén et al. 2009). Therefore, it is important to select conditions of alkaline hydrolysis that will yield the high-molecular-weight protein fractions, capable of film formation (Singh et al. 2017).

The aim of the study was to assess the statistically significant effect of input variables in the alkaline hydrolysis of keratin from white chicken feathers, carried out at room temperature using sodium hydroxide, on the process yield, molecular weight of the peptides obtained. The effects of the volume ratio of 1M NaOH to the feather mass, the hydrolysis time, and the shaking speed of the reaction mixture were statistically analyzed. The pretreatment process of feathers and the purification process of hydrolysates obtained were carried out according to typical methods reported by other authors (Schrooyen et al. 2000; Singh et al. 2017; Nakamura et al. 2002; Sinkiewicz et al. 2017); however, with some modifications. Namely, the processes of degreasing feathers with organic solvents and grinding feathers as well as dialysis and freeze-drying of hydrolysates obtained were omitted. Skipping these processes allowed eliminating the use of environmentally harmful reagents, to shorten the processing time, and to reduce energy consumption. All these steps together have contributed to making the process more environmentally friendly.

To the best our knowledge, no research has been conducted so far on a statistical analysis of the process conditions of alkaline hydrolysis of keratin from white chicken feathers. In our opinion, this allows for a precise optimization of the method in the context of obtaining protein fractions useful for the formation of biodegradable films.

## Materials and methods

### Materials

White chicken feathers were supplied by a local poultry slaughterhouse (Drobful, Poland). NaOH (POCH, Poland) was used to carry out the hydrolysis. DTT and Coomassie brilliant blue R-250 (Fluka, USA), SDS (Merck, Germany), 30% solution of acyrlamide/bis-acrylamide, ammonium persulfate, bromophenol solution, glycine, hydrochloric acid, TEMED, and trizma base (Sigma-Aldrich, USA), acetic acid, glycerol and methanol (Chempur, Poland), ammonium perfulfate, and prestained protein ladder PageRuler Plus (Thermo scientific, USA) were applied for determination of protein molecular weight. Sulfuric acid and Tashiro’s indicator (POCH, Poland), selenium catalyst (Chempur, Poland), boric acid, and hydrochloric acid (Stanlab, Poland) were used to determine the protein content by the Kjeldahl method.

### Pretreatment of feathers

To increase the hydrolysis yield, the pretreatment of feathers was performed by removing impurities and lipid compounds that naturally cover the feathers. This step has been simplified compared to the previous one (Sinkiewicz et al. 2017). Briefly, whole feathers were washed in warm water with detergent until impurities and lipids were removed, dried at 50°C for 24 h, and then cut into 2–3 cm long filaments.

### Alkaline hydrolysis

The hydrolysis process was carried out at room temperature to prevent strong hydrolysis of the peptide bonds and to obtain the high-molecular-weight protein fractions (Mokrejs et al. 2011). The pure defatted feathers were shaken (Thermo Forma 420 Orbital Shaker, Thermo Scientific, USA) at 150, 175, or 200 rpm for 24, 16, or 32 h, respectively, with distilled water in a ratio of 1:10 to the mass of feathers during the first hour, and next, 1M NaOH was added, and shaking was continued. The hydrolysates obtained were centrifuged at 5000 rpm for 15 min (MPW-350R Centrifuge, MPW Med. Instruments, Poland) to get rid of insoluble feather residues and filtered. The filtrate hydrolysates were neutralized with 1M HCl and used for analyses.

### Determination of keratin hydrolysis yield

The total nitrogen content in the dry matter of pure feathers and in the hydrolysates obtained was determined by the Kjeldahl method and then converted into the total protein content by using a literature conversion factor of 5.71 (AOAC 1990; Grazziotin et al. 2006). The hydrolysis yield was calculated based on the results of the total protein content in feathers and in hydrolysates:

1 $$Y\left[\%\right]=\frac{total\;protein\;content\;in\;hydrolysates\;\left[g\right]}{total\;protein\;content\;in\;feathers\;\left[g\right]}\cdot100\%$$

### Determination of solubility properties of hydrolysates

The possibility of using keratin hydrolysates in the film preparation depend not only on the hydrolysis yield, but also on their functional properties, such as solubility at different pH. To determine the solubility of hydrolysates obtained, the pH of each sample of hydrolysate suspension was adjusted to 2–7 by using 1M HCl, and the resulting turbidity was measured using a spectrophotometer (Spectroquant® Pharo 300, Merck, Germany) at 280 nm. The measurements were taken in triplicate, and results, expressed as mean values, were presented in the form of a plot of absorbance versus pH. Then, insoluble protein was precipitated from hydrolysate (the highest turbidity) with 1M HCl, filtered, dried, and weighed. The soluble protein content in the hydrolysates was determined at g/100 mL of hydrolysate.

### Determination of the molecular weight of peptides by sodium dodecyl sulfate–polyacrylamide gel electrophoresis (SDS-PAGE)

To determine the molecular weight of the peptides contained in the keratin hydrolysates, SDS-PAGE was performed according to the method of Laemmli (1970). The samples of 40 mg of dialyzed and then freeze-dried hydrolysate were mixed with 1 mL water, loaded onto each lane for comparison, resolved on a 12% separation gel, and stained with 0.25% (w/v) Coomassie brilliant blue. A marker having a molecular weight of 10-250 kDa was used as a standard.

### Process optimization using designed experiment

**Fig. 1:**
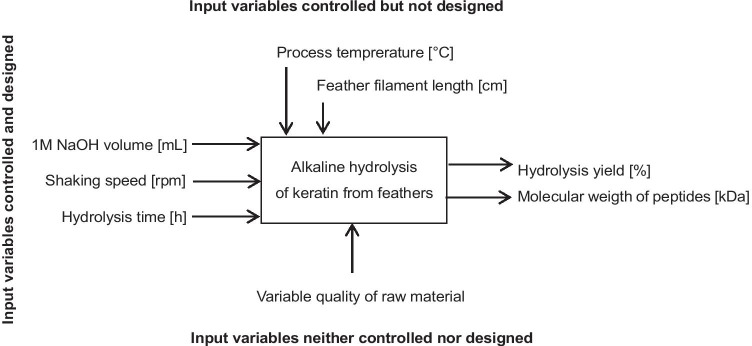
Black box model for keratin hydrolysis

The purpose of using the statistical analysis was to select the parameters of key importance in the process of alkaline hydrolysis. The hydrolysis process was designed using a black box model (Fig. 1). Three input variables for this process were selected, which were both controlled and designed: (i) the ratio of the 1M NaOH volume to the feathers mass [v/w], (ii) the hydrolysis time [h], and (iii) the shaking speed of the reaction mixture [rpm]. The ratio of the volume of 1M NaOH to the mass of feathers was 3:1, 6:1, and 9:1 [v/w], corresponding to 75, 150, and 225 mL of 1M NaOH, respectively. The hydrolysis time was 16, 24, and 32 h, and the shaking speed of the reaction mixture was 150, 175, and 200 rpm.

Performing the hydrolysis for three different volumes of 1M NaOH, three different hydrolysis times, and three different shaking speeds would require 27 combinations of these parameters for only one repetition. To determine which process conditions has a significant effect on the hydrolysis yield and the resulting protein size, the method of statistically significant effect was applied at the design step. Latin square was used for this purpose. This method reduced the numbers of trials needed to be performed from 27 to 9, thus, reducing the cost and time of the study by three times. The Latin square method meets all the conditions of the research model, such as randomization, number of input variables, and levels of variation, and allows easy and quick application without the need for specialized software (de Winter and Dodou 2017; Korzyński 2013).

The left side of Table 1 shows a general scheme of the randomized Latin square. The input variables were randomly recorded at three levels of variation (*n*=3), from −1 to 1 for variables *x*_1_ and *x*_2_, in columns and rows, respectively, and from A to C for variable *x*_3_, located inside the square. The right side of Table 1 presents the Latin square for the alkaline hydrolysis, filled with the process conditions, that was created based on the general scheme. After determining the hydrolysis yield and the molecular weight of peptides obtained, the results will be inserted into the Latin square, and for each of the input variables tested, the Fisher-Snedecor statistics, called the empirical F value, will be calculated (de Winter and Dodou 2017; Korzyński 2013). Results of the statistical test will answer the question of which input variables in the alkaline hydrolysis process of keratin from feathers had a significant effect on the keratin yield.

**Table 1:** Permutated Latin square with three levels of variations filled with conditions of alkaline hydrolysis process

Levels of the variability for the input variables		Variable *x*_1_					Volume of 1M NaOH [mL]		
		−1	0	1			75	150	225
Variable *x*_2_	−1	A	C	B	Hydrolysis time [h]	16	150	200	175
	0	C	B	A		24	200	175	150
	1	B	A	C		32	175	150	200

−1, 0, 1—three levels of the variability for the input variable *x*_1_, volume of 1M NaOH [mL], and x_2_, hydrolysis time [h]

A, B, C—three levels of the variability for the input variable *x*_3_, shaking speed of the reaction mixture [rpm]

## Results and discussion

### Effect of input variables on keratin hydrolysis yield

The average total protein content in the dry matter of feathers was 87.9 ± 0.58% and was similar to that reported by other authors. According to Kumar et al. (2012), it is more than 85%, and according to Saravanan and Dhurai (2012), it is 90%.

**Fig. 2:**
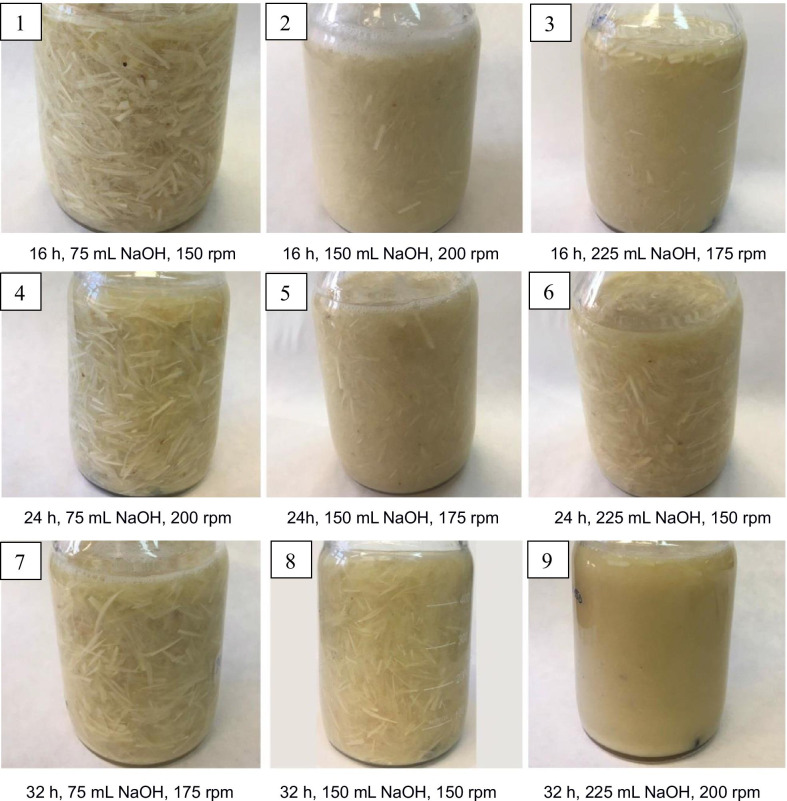
Photos of reaction mixtures after hydrolysis (please, compare the captions under the drawings with the names of columns 1–4 in Table 2)

The highest yield of keratin isolation carried out at room temperature was achieved when the volume of 1M NaOH was the highest and was 225 mL, regardless of the other process parameters (Table 2). This yield was ca. 24, 37, and 41% after 16, 24, and 32 h hydrolysis time, respectively. Figure 2 presents the photos of reaction mixtures taken before the filtration step. Undissolved feather residues were found in all trials, except for sample no. 9, in which the highest input variable parameters were used, i.e., volume of 1M NaOH, 225 mL, the hydrolysis time, 32 h, and shaking speed of the reaction mixture, 200 rpm. Taking into accounts the results of the hydrolysis yield (Table 2), it can be seen that the lower amount of undissolved residue (Fig. 2), the higher the hydrolysis yield was achieved. According to Nomura et al. (2007), the yield of the feather hydrolysis process carried out at room temperature ranges from 30 to 70%, depending on the process time and the alkali concentration used. The much higher hydrolysis yield realized by these authors was probably due to a 3–10 times higher ratio of 1 M NaOH volume to feather mass than that used in presented study. Our previous study revealed that by conducting the alkaline hydrolysis not at room temperature but at 70°C, at the ratio of 1M NaOH to feather mass of 10:1 [v/w] and for a period of 1 h and 15 min, keratin can be obtained with yield of ca. 29% (Sinkiewicz et al. 2017). Thus, the results presented in Table 2 indicate that by lowering the process temperature but running the process for a longer period of time, the higher process yield can be obtained.

**Table 2:** Effect of input variables on keratin hydrolysis yield

Hydrolysate no.	1M NaOH [mL]	Hydrolysis time [h]	Shaking speed [rpm]	Total protein content [g/100 mL]	Hydrolysis yield [%]
1	75	16	150	2.49 ± 0.19	11.33 ± 0.87
2	150	16	200	3.33 ± 0.07	15.17 ± 0.33
3	225	16	175	5.24 ± 0.54	23.84 ± 2.44
4	75	24	200	2.52 ± 0.16	11.45 ± 0.74
5	150	24	175	3.49 ± 0.20	15.90 ± 0.90
6	225	24	150	8.12 ± 0.08	36.96 ± 0.36
7	75	32	175	2.85 ± 0.15	12.96 ± 0.66
8	150	32	150	5.06 ± 0.07	23.04 ± 0.34
9	225	32	200	9.02 ± 0.32	41.05 ± 1.46

The results of hydrolysis yield were inserted into the relevant Latin square fields as shown in Table 3. The calculations of the significance of the effect of one input variable on the hydrolysis yield, namely the volume of the 1M NaOH, are presented below. The calculations for the two other input variables, the hydrolysis time and the shaking speed, were performed analogously.

**Table 3:** The randomized Latin square filled with the results of hydrolysis yield

Levels of input variability		Volume of 1M NaOH [mL]		
		75	150	225
Hydrolysis time [h]	16	11.33	15.17	23.84
	24	11.45	15.90	36.96
	32	12.96	23.04	41.05

To determine the empirical *F* value, in the first step, the results from each of the three columns (*C*_1_, *C*_2_, *C*_3_), indicated the volume of 1M NaOH used (75 mL, 150mL, 225 mL), were summed:

2 $${\displaystyle \begin{array}{c}\begin{array}{c}{C}_i=\sum\limits_{i=1}^P{y}_i\\ {}{C}_1=11.33+11.45+12.96=35.74\end{array}\\ {}{C}_2=15.17+15.90+23.04=54.11\\ {}{C}_3=23.84+36.96+41.05=101.85\end{array}}$$

In the second step, the sums of the results from the columns were squared and divided by numbers of levels of variability (*n*=3), obtaining the arithmetic mean of the squares of these quantities (*C*^2^):

3 $${\displaystyle \begin{array}{c}{C}^2=\frac{1}{n}\sum\limits_{i=1}^P{C_i}^2\\ {}{C}^2=\frac{35.74+54.11+101.85}{3}=4\ 859.55\end{array}}$$

Next, the square of arithmetic average of the sum over all experimental results (Δ*y*), for *n*=3 was calculated:

4 $${\displaystyle \begin{array}{c}\Delta y=\frac{1}{n^2}{\left(\sum\limits_{i=1}^{p^2}{y}_i\right)}^2\\ {}\Delta y=\frac{{\left(\mathrm{11..33}+15.17+23.84+11.45+15.90+36.96+12.96+23.04+41.05\right)}^2}{9}=4\ 083.21\end{array}}$$

The *C*^2^ value was decreased by Δ*y* value, obtaining the sum of squares for the columns (*S*_C_). In the next step, the Δ*y* value was subtracted from the sum of squares of all results from the Latin square, obtaining the overall sum of squares of all experimental results (*S*):

5 $${\displaystyle \begin{array}{c}{S}_C={C}^2-\Delta y\\ {}{S}_C=4\ 859.55-4\ 083.21=776.34\end{array}}$$

6 $${\displaystyle \begin{array}{c}S=\sum\limits_{i=1}^{p^2}{y_i}^2-\Delta y\\ {}S=\left({11.33}^2+{15.17}^2+{23.84}^2+{11.45}^2+{15.90}^2+{36.96}^2+{12.96}^2+{23.04}^2+{41.05}^2\right)-4\ 081.23=977.49\end{array}}$$

To calculate the sum of squares for the experimental error (*S*_E_), the *S*_C_ values as well as *S*_R_ and *S*_L_ values, representing the sum of squares for the rows and for the letters, respectively, were subtracted from the value of *S*:

7 $${\displaystyle \begin{array}{c}{S}_E=S-{S}_R-{S}_C-{S}_L\\ {}{S}_E=977.49-118.99-776.34-64.95=17.21\end{array}}$$

The degrees of freedom *f* for the columns (*f*_c_), the rows (*f*_R_), and the letters (*f*_L_) and the degrees of freedom for the error (*f*_E_) were:

$${f}_C=n-1$$

$${f}_R=n-1$$

$${f}_L=n-1$$

$${f}_E=\left(n-1\right)\left(n-2\right)$$

$${f}_R={f}_C={f}_L=3-1=2$$

$${f}_E=\left(3-1\right)\left(3-2\right)=2$$

In the next step, the variance value for columns (*S*_C_^2^) and error (*S*_E_^2^) was calculated by dividing the sum of their square by the number of their degrees of freedom (*f*_C_ or *f*_E_).

8 $${\displaystyle \begin{array}{c}{S_C}^2=\frac{S_C}{f_C}\\ {}{S_C}^2=\frac{17.21}{2}=8.6\end{array}}$$

9 $${\displaystyle \begin{array}{c}{S_E}^2=\frac{S_E}{f_E}\\ {}{S_E}^2=\frac{17.21}{2}=8.60\end{array}}$$

To calculate the variance quotients, denoting the empirical *F* values, the variance for the columns (*S*_C_^2^) was divided by the variance for the error (*S*_E_^2^). This calculated empirical *F* value was then compared with a critical *F*_cr_ value of Fisher-Sendecor distribution for a significance level of *α*=0.05 and the number of degrees of freedom *f*_1_ = *f*_C_ = *f*_R_ = *f*_L_ and *f*_2_ = *f*_E_ as equal 19.

10 $${\displaystyle \begin{array}{c}{F}_C=\frac{{S_C}^2}{{S_E}^2}\\ {}{F}_C=\frac{388.17}{8.6}=45.11\end{array}}$$

11 $$\begin{array}{c}\begin{array}{c}F_{cr}=F_{\left(\alpha;f_1;f_2\right)}\\F_{cr}=F_{\left(0,05;2;2\right)}=19\end{array}\\F_C(45.11)\geq F_{cr}(19)\end{array}$$

If the empirical *F* value was ≥ *F*_cr_, then the effect of the input variable on the outcome factor variable will be considered significant with 95% confidence level. The *F*_C_ was equal 45.11 so the effect of the volume of 1M NaOH solution on the hydrolysis yield is considered significant.

The results of the other empirical *F* value, *F*_R_ and *F*_L_ for each input variable, were sequential: 6.91 for the hydrolysis time, 3.77 for the shaking speed. These results are lower than *F*_cr_ =19 thus should be considered as insignificant on the hydrolysis keratin yield in the ranges tested.

### Solubility properties of hydrolysates

**Fig. 3:**
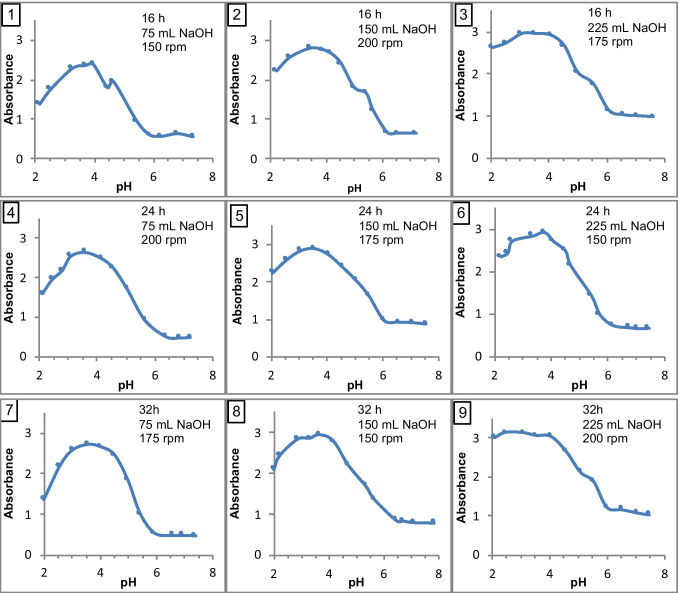
The plots of absorbance versus pH indicating solubility properties of hydrolysates

The results presented in Figure 3 show that the least soluble proteins were in a solution of pH 3.4–4.0, i.e., the pH close to the isoelectric point (pI) of keratin. According to Bragulla and Homberger (2009), pI of reduced keratin determined by the electrophoretic method is in the range of 4.9–5.4. The differences in the pI values obtained were probably due to the use of a different method of keratin isolation and the presence in the hydrolysate obtained of a mixture of polypeptides with different molecular weight and different pI. The method of pI determination could also have influenced the results. In the presented study, peptides were precipitated from a strongly alkaline solution using HCl, which led to the high salinity of the mixture. Under condition of high NaCl content, the proteins are deprived of their water envelope. Then, the strength of intermolecular hydrophobic interactions increases, which leads to the precipitation of proteins from the solution and, consequently, to a decrease in their solubility, and thus a shift of the pI towards lower values.

**Fig. 4:**
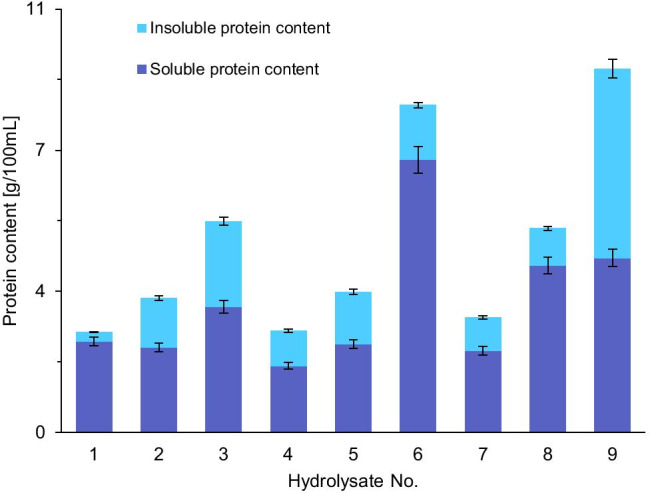
The content of soluble and insoluble proteins in the hydrolysates obtained

The content of insoluble protein [g/100mL] in the hydrolysates obtained, founded after its precipitation and drying, was compared with the content of total protein [g/100mL] (Fig. 4). These results clearly prove that in all hydrolysates the soluble protein fraction was predominant.

### Determination of the molecular weight of peptides by SDS-PAGE

**Fig. 5:**
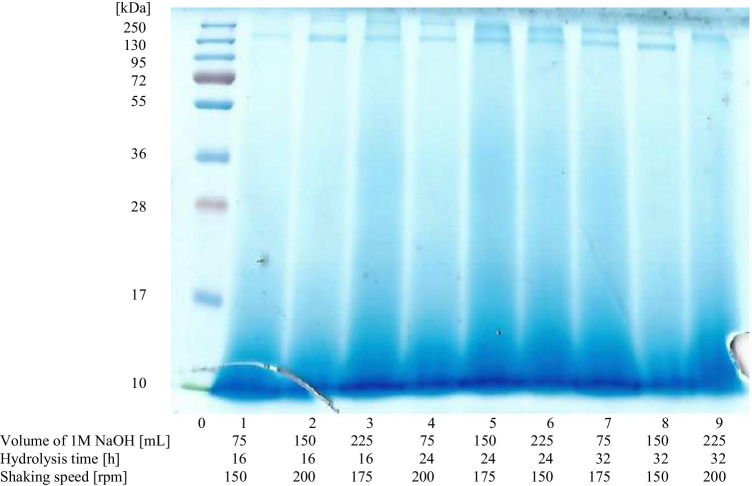
SDS-PAGE profiles of hydrolysates (1–9) with the protein ladder on lane 0. The numbers of hydrolysates are in accordance with Table 2

Electrophoretic profiles of hydrolysates obtained revealed subtle differences. While two peptide fractions of 130 and 250 kDa molecular weights were dominated in all hydrolysates, in that of No. 9, obtained under the most extreme conditions (the volume of 1M NaOH, 225 mL, the hydrolysis time, 32 h, the shaking speed of the reaction mixture, 200 rpm), the peptide fraction of 130 kDa was not observed (Fig. 5). Moreover, the profiles of all hydrolysates indicated the presence of peptides with the molecular weight lower than 10 kDa. According to Wang et al. (2016b), the molecular weight of α-keratin ranges in size from 40 to 68 kDa, and that of β-keratin from 10 to 22 kDa. Thus, presence of peptide fractions of much higher molecular weights in the hydrolysates obtained can indicate an incomplete rupture of peptide bonds during hydrolysis process. However, the results of other authors indicate that alkaline hydrolysis of keratin leads to peptides of various sizes, depending on the process conditions. Nomura et al. (2007) using alkaline hydrolysis with 1M NaOH received peptides of 8–13 kDa, but at the volume ratio of alkali to the feathers mass 3–10 times higher than that used in the presented work. Song et al. (2013) obtained fractions with a molecular weight of 5.8 kDa after alkaline hydrolysis of feather keratin. These differences in molecular weight can be explained by the extraction conditions, such as hydrolysis time, volume, and concentration of alkaline and shredding treatments such as blending and homogenization of feathers or reaction mixture, which were different in each study. The results of SDS-PAGE analysis indicated also different concentrations of peptides present in each of the hydrolysates as the intensity of individual bands in their profiles varied (Fig. 5). The greatest intensity of the bands was observed in the samples whose hydrolysis proceeded with the highest volume of 1M NaOH (lane 3, 6, and 9), regardless of other process conditions, such as hydrolysis time and shaking speed of the reaction mixture.

## Conclusions

The randomized Latin square statistical program allowed to assess the significance of the influence of three input variables, with three levels of variability, on the final result of alkaline hydrolysis of soluble keratin from chicken feathers, limiting the number of trials from 27 to 9 necessary to perform, which reduced research cost and time. The use of the Latin square has made it possible to determine which variables in the process of alkaline hydrolysis of feather keratin had a significant impact on the protein yield. The hydrolysis yield was significantly influenced by the volume ratio of 1M NaOH to the mass of feathers, and the influence of hydrolysis time and shaking speed of the reaction mixture in the tested ranges on this yield were insignificant. Alkaline hydrolysis of feather keratin allowed obtaining a protein hydrolysate soluble in the wide pH range. SDS-PAGE electrophoreogram analysis showed that the conditions of alkaline hydrolysis in the tested ranges had little effect on the size of the keratin peptides obtained.
